# Differences in virulence gene expression between human blood and stool *Campylobacter coli* clade 1 ST828CC isolates

**DOI:** 10.1186/s13099-019-0322-9

**Published:** 2019-08-01

**Authors:** Cecilia Johansson, Anna Nilsson, René Kaden, Hilpi Rautelin

**Affiliations:** 1 Clinical Microbiology, Department of Medical Sciences, Uppsala University, Uppsala, Sweden; 2 Present Address: Department of Ecology, Swedish University of Agricultural Sciences, Uppsala, Sweden

**Keywords:** *Campylobacter coli*, Clade 1, ST828CC, Bacteraemia, Blood, Stool, Adhesion, Virulence, *cdt*

## Abstract

**Background:**

*Campylobacter* colonise the gastrointestinal tract of warm-blooded animals and are major enteropathogens in humans. *C. coli* is less common than *C. jejuni* and accounts for about 10% of the total number of *Campylobacter* infections although the two species seem to share many virulence determinants. *Campylobacter* bacteraemia is rare, estimated to occur in less than 1% of the infections, and the exact mechanisms regulating the progression of the infection from the gastrointestinal tract to the blood stream are unclear. Here, we looked at the contribution of *C. coli* to *Campylobacter* infections and further compared various virulence traits in *C. coli* clade 1 blood and stool isolates.

**Results:**

We assessed the numbers of *C. jejuni* and *C. coli* among typed isolates in the PubMLST database and found that *C. coli* accounted for 25.9% of blood isolates, but only 8.9% of the stool isolates. Phylogenetic analysis of 128 *C. coli* clade 1 whole genome sequences deposited to NCBI revealed no specific clustering of the human blood, stool or animal isolates. Of the six *C. coli* isolates chosen for phenotypic analyses, stool isolates adhered significantly better to human HT-29 colon cancer cells than the blood isolates, while there was no difference in induced IL-8 levels between the isolates. Furthermore, the stool isolates had two- to fourfold higher RNA expression levels of the *flpA*, *ciaB, iamA* and *cdt* virulence genes than the blood isolates. Finally, we looked at the gene structure of the *cdtA, B* and *C* toxin genes and found numerous nucleotide additions and deletions disrupting the open reading frames. In contrast to 58% isolates of animal origin, only 38% and 32% of human blood and stool isolates, respectively, had all three *cdt* genes intact, a prerequisite to produce functional toxins.

**Conclusions:**

This study reveals interesting differences between *C. coli* clade 1 isolates of human and animal origin on one hand, and also between human blood and stool isolates, on the other. The results suggest that *C. coli* might downregulate and/or inactivate various virulence determinants as the isolates pass from the animal host to the human gastrointestinal tract and enter the human blood stream.

**Electronic supplementary material:**

The online version of this article (10.1186/s13099-019-0322-9) contains supplementary material, which is available to authorized users.

## Background

*Campylobacter* colonise the gastrointestinal tract of warm-blooded animals, such as poultry and wild birds, ruminants, pigs, cats and dogs but rarely cause any symptoms in animal hosts. However, when transmitted to humans, *Campylobacter* cause gastroenteritis worldwide [1]. It has been estimated that about 1% of Europeans develop campylobacteriosis every year [2], and *Campylobacter* have been the most commonly reported bacterial enteropathogen since 2005 in the European Union, with over 246,000 reported cases in 2016 [3].

*Campylobacter* infection usually develops one to 5 days after ingestion of the bacteria, with typical symptoms including fever, headache, vomiting and abdominal pain in addition to watery or even bloody diarrhoea [4]. Known post-infection complications are reactive arthritis [5], irritable bowel syndrome [6], and the severe autoimmune demyelinating neuropathy Guillain-Barré syndrome [7], which substantially increase the burden of the disease, which in the USA has been estimated to be 22 500 disability-adjusted life-years [8].

*Campylobacter coli* is less common than *C. jejuni* as a human pathogen and seems to account for about 10% of the total number of *Campylobacter* infections [9]. *C. coli* infection cannot be distinguished from that of *C. jejuni* on the basis of the clinical picture [9]. *C. coli* isolates cluster into three clades [10–12], where those of clade 1 are mainly clinical and animal isolates, including the clonal complexes of ST828CC and ST1150CC [13], whereas clade 2 and clade 3 lack clonal complex structure and mainly contain isolates of environmental origin [10, 12].

*Campylobacter* bacteraemia is rare, estimated to occur in less than 1% of the total number of *Campylobacter* infections [14, 15], and then mainly in the elderly and in patients with immune deficiencies or other underlying conditions [15, 16], although young and otherwise healthy individuals have also been shown to be affected [17, 18]. *C. jejuni* is the predominant pathogen among campylobacteriosis cases and most commonly isolated from blood while *C. coli* accounts for around 10% of bacteraemia cases [14, 17, 19].

Campylobacteriosis is considered a multifactorial process in which bacterial properties such as motility, adhesion and invasion seem to be important for virulence [20], and mutational studies in *C. jejuni* have identified several virulence genes implicated in these processes. Adhesion is thought to be initiated by CadF and FlpA, which are bacterial adhesins known to bind fibronectin on the host cell surface [21–24]. Secreted *Campylobacter* invasion antigens, such as CiaB, have been shown to thereafter be needed for efficient invasion into the cells [25, 26]. Also, the invasion-associated marker, IamA, is considered to play a role in invasion, as it has been reported to be present in the majority of invasive, but only in the minority of non-invasive, *Campylobacter* isolates [27]. The cytolethal distending toxin, CDT, is well-known among *Campylobacter* and also produced by many other bacterial pathogens, such as *Salmonella* and *E. coli* (reviewed in [28]). CDT consists of three subunits; the enzymatically active B subunit and the regulatory A and C subunits. The DNase activity of CdtB induces double-strand breaks in the host chromosomes, eventually leading to cell cycle arrest at the G2/M interphase. CDT might also contribute to inflammation as it has been shown to induce IL-8 secretion from INT-407 cells through its binding to host cell membranes [29].

Why some *Campylobacter* isolates are more invasive than others and enter the bloodstream is not well understood. In this study, we compared genotypic and phenotypic characteristics of *C. coli* clade 1 blood and stool isolates and found that, although they are very similar genetically, they showed differences in gene expression levels of several virulence factors.

## Results

### *Campylobacter coli* in the PubMLST/Campylobacter database

**Fig. 1:**
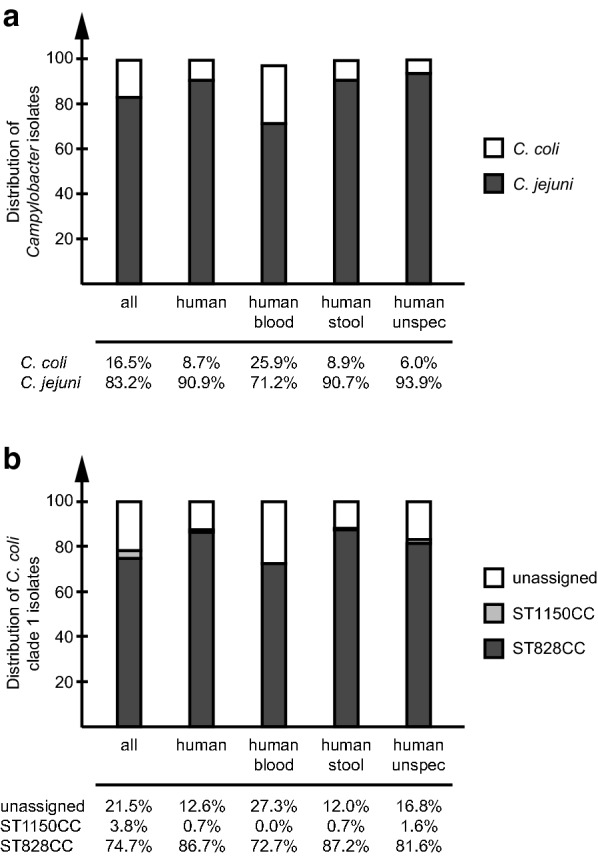
Distribution of *Campylobacter jejuni* and *C. coli* isolates in PubMLST. **a** Distribution of *C. jejuni* and *C. coli* isolates among all isolates and those of human, blood, stool and unspecified human origin. **b** Distribution of *C. coli* clade 1 isolates belonging to ST828CC, ST1150CC or unassigned to any clonal complex

To better understand the contribution of *C. coli* to *Campylobacter* infection and bacteraemia, the number of *C. jejuni* and *C. coli* isolates from human and animal sources were retrieved from PubMLST (https://pubmlst.org/campylobacter/; last accessed Feb 8th, 2019) (Additional file 1: Table S1). Of all 71,559 *Campylobacter* isolates in the jejuni/coli database, *C. coli* accounted for 16.5%, but only 8.7% when counting isolates from exclusively human sources (Fig. 1a). However, when looking at the human blood and stool isolates separately, there was a clear difference in the distribution, where *C. coli* accounted for 25.9% of blood isolates, but only 8.9% of the stool isolates (Fig. 1a). For *C. jejuni*, this distribution was reversed with a higher percentage for the stool isolates than for the blood isolates (Fig. 1a).

Within *C. coli*, ST828CC comprised 74.7% of all MLST-typed *C. coli* isolates and 86.7% of all *C. coli* isolates from human sources (Fig. 1b). When comparing *C. coli* human blood and stool isolates, there was a lower fraction of ST828CC among the blood isolates (72.7%) compared to that of the stool isolates (87.2%). Moreover, there were no blood isolates of ST1150CC in the database but instead a large percentage of blood isolates unassigned to a clonal complex (Fig. 1b).

### Phylogenetic analyses

Whole genome sequences for human blood, human stool and animal *C. coli* isolates belonging to ST828CC (Additional file 1: Table S2) were retrieved from GenBank, National Center for Biotechnology Information (http://www.ncbi.nlm.nih.gov/GenBank/index.html; last accessed Feb 1st, 2019). To this dataset, we added three whole genome sequenced blood isolates (B5, B49 and B63) that were sequenced in this study and three previously sequenced stool isolates (F3, F4 and F8) [30]. All isolates were confirmed to belong to ST828CC by a sequence query of the whole genome sequences against the PubMLST database (https://pubmlst.org/campylobacter/; last accessed Feb 25th, 2019). Phylogenetic analyses of all 126 included ST828CC whole genome sequences together with the clade 1 reference strains RM2228 and LMG 6440, the clade 3 human stool isolate 76339 and previously clade 2- and 3-assigned water isolates [12] confirmed the clade assignment of the isolates (Additional file 2: Fig. S1). However, there was no specific clustering of either the human blood and stool or animal isolates.

### Adhesion and IL-8

**Fig. 2:**
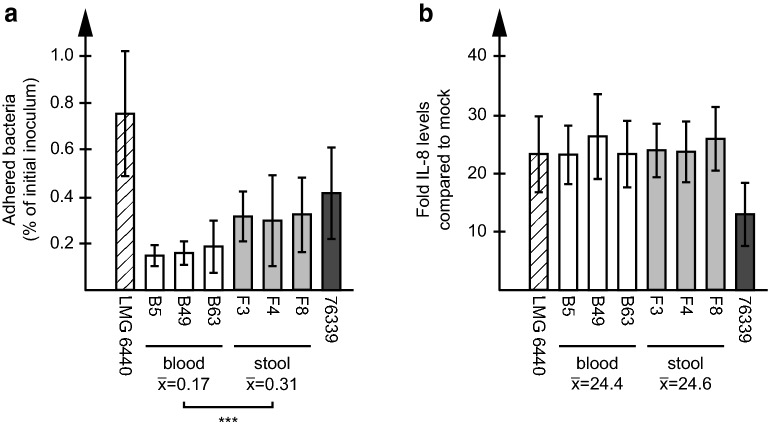
Infection of human HT-29 colon cancer cells. HT-29 cells were infected with *C. coli* clade 1 blood (B5, B49, B63) and stool (F3, F4, F8) isolates at a MOI of 100 for indicated time periods. The LMG 6440 reference strain and the clade 3 stool isolate 76339 were included for comparison. **a** Cells were harvested and lysed, and adhered bacteria were quantified using qPCR and expressed as percentage of the starting inoculum. The graph shows mean and standard deviation of at least two independent infection experiments. The means of the blood and stool isolates, respectively, are shown below the graph. A significant difference between blood and stool isolates is indicated with asterisks (p < 0.001). **b** IL-8 levels in the cell culture media were measured using ELISA and expressed as fold increase over mock-infected cells. The graph shows mean and standard deviation of at least two independent infection experiments. The means of the blood and stool isolates, respectively, are shown below the graph

To phenotypically compare *C. coli* clade 1 ST828CC human blood and stool isolates, we chose three blood (B5, B49 and B63) and three stool (F3, F4 and F8) isolates for further analyses. The adhesion to human HT-29 colon cancer cells was assessed using our previously established in vitro infection model [31]. For comparison, we used the LMG 6440 reference strain and the *C. coli* clade 3 human stool isolate 76339 [32]. The adhesion levels increased with time (data not shown) but were lower for clade 1 blood and stool isolates than for both the clade 1 reference strain LMG 6440 and the clade 3 stool isolate 76339 (Fig. 2a). The stool isolates adhered significantly better than blood isolates. Both blood and stool isolates induced IL-8 to around 20-fold over that of mock-infected cells, similar to that of LMG 6440 but higher than that shown for 76339 (Fig. 2b).

### Differences in expression of virulence genes between *C. coli* blood and stool isolates

**Fig. 3:**
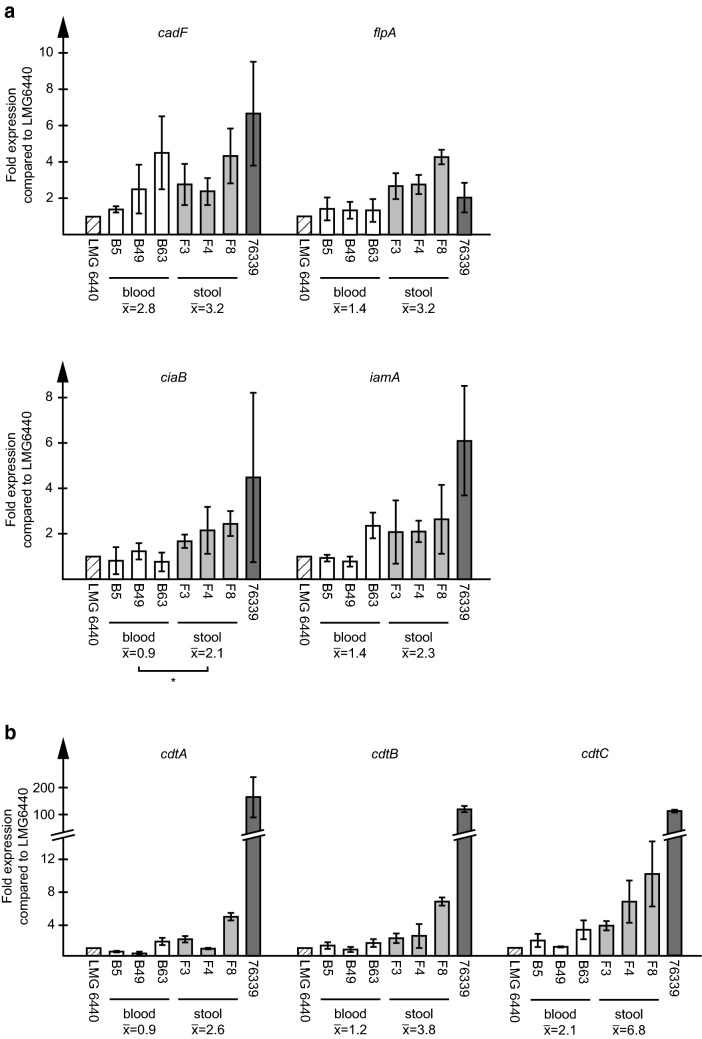
Virulence gene expression. **a**, **b** RNA expression of selected putative virulence genes was analysed using RT-qPCR. Data are expressed as relative to the LMG 6440 reference strain for each gene and shown as mean and standard deviation of at least two independent RNA preparations. The means of the blood (B5, B49, B63) and stool (F3, F4, F8) isolates, respectively, are shown below each graph. A significant difference between blood and stool isolates is indicated with an asterisk (p < 0.05)

We previously showed differences in expression levels of putative virulence genes between *C. coli* clades 1, 2 and 3 [30]. Here, we wanted to see whether there were any differences between clade 1 human blood and stool isolates. The clade 1 stool isolates had higher expression levels of *flpA*, *ciaB* and *iamA* than the blood isolates, while there was no significant difference in *cadF* expression levels (Fig. 3a). Also for the *cdtA*, *cdtB* and *cdtC* genes, there was a difference between the blood and stool isolates where the stool isolates had approximately three times higher expression levels than the blood isolates (Fig. 3b).

### Genomic analyses of virulence genes

Nucleotide sequences for the *cadF*, *flpA*, *ciaB* and *iamA* virulence genes were retrieved from the whole genome sequences of the human blood (B5, B49 and B63) and stool (F3, F4, F8) isolates together with the RM2228 and LMG 6440 reference strains. In silico translation of the open reading frames (ORFs) revealed very few differences between amino acid sequences of the blood and stool isolates (data not shown).

**Fig. 4:**
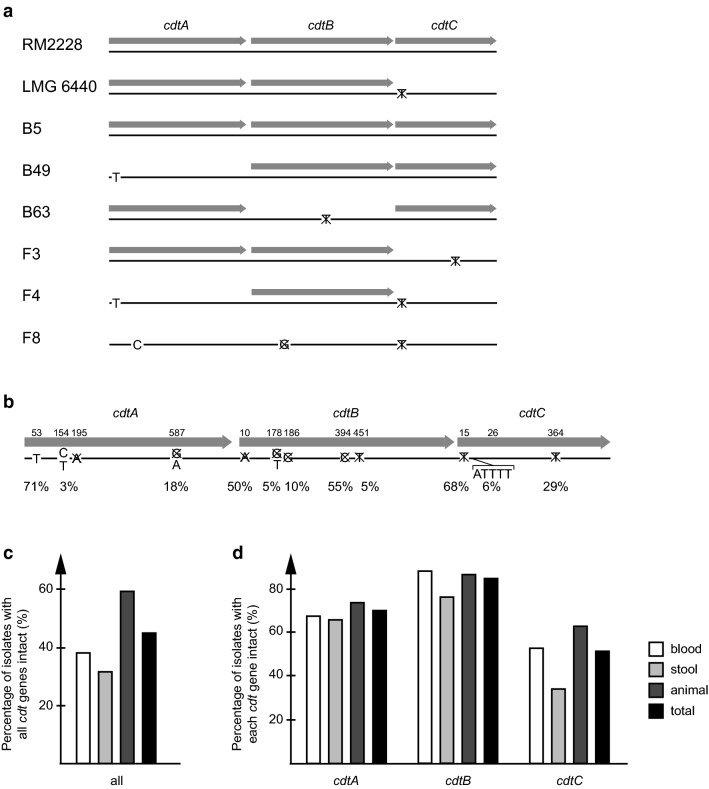
In silico analyses of *cdtA, B* and *C* gene structures in *C. coli*. **a** Schematic picture showing intact *cdtA, B* and *C* ORFs in the blood (B5, B49 and B63) and stool (F3, F4 and F8) isolates as well as the reference strains RM2228 and LMG 6440 as grey boxed arrows. Letters denote nucleotide insertions, while crossed-over letters denote deletions in the nucleotide sequences (black lines). **b** All identified mutations leading to disrupted ORFs in the *cdt* genes of the 128 clade 1 sequences that were used in the phylogenetic analysis. Numbers above the ORFs indicate the position of each mutation, letters denote nucleotide insertions, crossed-over letters denote nucleotide deletions and the prevalence of each specific mutation is indicated as a percentage. Percentage of the isolates in each group (blood, n = 34; stool, n = 38; and animal, n = 54) with all (**c**) and each (**d**) *cdt* ORF intact, respectively

For the *cdt* genes, however, the same analysis revealed several nucleotide insertions or deletions resulting in frame shifts and premature stop codons in the gene sequences, eliminating the theoretical ability to translate and produce full length proteins and functional toxins (Fig. 4a).

Only the blood isolate B5 and RM2228 had all three *cdt* ORFs intact, while the other isolates had at least one ORF disrupted (Fig. 4a). To see whether these *cdt* mutations were a general phenomenon, the *cdt* gene analysis was extended to include all the *C. coli* ST828CC isolates from human blood, stool or animal samples used in the phylogenetic analyses (Additional file 1: Table S2). The same trend of disrupted *cdt* ORFs by insertions or deletions was found. Figure 4b shows all identified disrupting mutations in each gene with the nucleotide position and prevalence of each event. All the three *cdt* genes had one highly prevalent mutation at the beginning of the gene. For almost all isolates included, each gene had only one disrupting mutation, except for *cdtB*, where 30% of the isolates with a disrupted *cdtB* ORF had both an A deletion at position 10 and a C deletion at position 394 (data not shown). In the human blood and stool isolate groups, 38% and 32% of all isolates had all *cdt* genes intact, respectively, while in the animal group, this number was substantially higher (58%, Fig. 4c). There was no big difference in the percentage of isolates in each group with intact genes for both *cdtA* and *cdtB*, while the percentage of intact *cdtC* was lower for the stool isolates compared to the blood and animal isolates (Fig. 4d).

## Discussion

The aim of this study was to compare various virulence traits in *C. coli* blood and stool isolates. First, we used the PubMLST/Campylobacter database to get an understanding of the contribution of *C. coli* to human infections. The percentage of 8.7% *C. coli* among all human isolates is in line with the reported percentage of 10% *C. coli* in human *Campylobacter* infections. However, when looking at *Campylobacter* isolates from all sources, the contribution of *C. coli* was higher (16.5%), suggesting that *C. coli* might be more prevalent among animal and environmental isolates. *C. coli* environmental isolates mainly belong to clades 2 and 3 and our earlier studies have identified several specific traits that would contribute to their survival in the environment [12, 30].

Surprisingly, when only looking at the human isolates in the PubMLST database, there was a substantially larger fraction of *C. coli* among the blood isolates (25.9%) compared to the stool isolates (8.9%). This can be interpreted in several ways. Either that *C. coli* might be better than *C. jejuni* to invade the blood stream and cause a more severe disease and that these numbers truly reflect different characteristics of the two species, or simply that there has been more interest to characterise *C. coli* blood isolates, as they are a less common cause of bacteraemia. Interestingly, a large fraction (27.3%) of the *C. coli* blood isolates were not assigned to any clonal complex, suggesting that these particular isolates might be genetically different from the commonly detected *C. coli* stool isolates.

We were further interested in whether human *C. coli* blood and stool isolates could show phenotypic and genetic differences that might explain the successful invasion of the blood stream. In our established in vitro infection model using HT-29 colon cancer cells, all *C. coli* blood and stool isolates were able to adhere to the cells and evoke an IL-8 response. The stool isolates adhered significantly better than the blood isolates at 3 h post infection while induced IL-8 levels were similar for all isolates tested. Neither did we see a difference in adhesion between *C. jejuni* blood and stool ST21CC isolates in our earlier study [31], suggesting that adherence potential rather varies on the isolate level than depending on isolation source and genetic origin.

Nucleotide and amino acid sequences of genes involved in adhesion and invasion, such as *cadF*, *flpA, ciaB* and *iamA*, were very similar for the *C. coli* blood and stool isolates. However, the genes did show differences in RNA expression levels between the two groups of isolates. *flpA*, which is involved in adhesion through its binding to fibronectin, and *ciaB* and *iamA*, which are involved in invasion, were expressed to higher levels in stool isolates than in blood isolates. For the *cdt* toxin genes *cdtA, B* and *C*, the differences between the blood and stool isolates were even larger, although the stool isolates varied in their expression levels. *C. coli* clade 1 stool isolates were earlier shown to have lower expression levels of several virulence genes compared to *C. coli* clade 2 and 3 water isolates [30]. All these results together highlight the importance of measuring actual expression levels of genes and not only looking at presence of the genes, as many studies show vague correlation between presence of virulence genes demonstrated by PCR and severity of disease. Interestingly, we also found several disruptive mutations in the *cdt* genes. The most intact gene was *cdtB*, which had a complete ORF in 84% of all isolates. This is expected since *cdtB* is the enzymatically active component of the toxin, and enzymes usually are more evolutionary conserved than regulatory genes. These types of mutations were not seen in any of the virulence genes involved in adhesion and invasion studied here or in any of the 80 *C. coli* clade 2 and 3 environmental isolates investigated in our previous study [30], excluding, in our opinion, the risk of this phenomenon being due to methodological errors during sequencing.

*Campylobacter* show great capability to adapt to different hosts and environments. The expression level differences of the virulence genes and the differences in *cdt* gene structure between isolates from different sources suggest that *C. coli* might be able to downregulate and/or inactivate selected virulence mechanisms once they established in the human gut but even more so after invading the blood stream. *C. jejuni* stool isolates of ST21CC, as compared to blood isolates, have been shown to possess larger amounts of accessory gene content, such as bacteriophage-derived integrated elements (CJIEs) and plasmids [31], possibly resulting in increased invasive potential.

Induction of cell cycle arrest in the small intestine by the CDT toxin would prevent the regeneration of the intestinal epithelium, which then might facilitate invasion. This particular activity would not be so important for bacterial survival and maintenance when the infection has already established in the gut and/or spread to the blood stream. It was earlier shown that *C. jejuni* human isolates produced much higher levels of the CDT toxin compared to *C. coli* human isolates [33], indicating that the CDT toxin might not be as important in *C. coli* virulence.

## Conclusions

In this study, we present interesting differences in virulence gene structure and expression levels between *C. coli* clade 1 blood and stool isolates. This data suggests that *C. coli* might downregulate and/or inactivate their virulence mechanisms once they have established the infection in the human gut and invaded the blood stream, and contributes to the understanding of *Campylobacter* pathogenesis.

## Methods

### Bacterial isolates

*Campylobacter coli* clinical isolates from blood [18] and stool [34] have been described previously. The *C. coli* clade 1 LMG 6440 (NCTC 11366) reference strain, which was originally isolated from a pig, was also included for comparison.

### Bacterial culture conditions

Bacteria were grown microaerobically (Oxoid Campygen, Thermo Fisher Scientific, Waltham, USA) at 42°C and routinely resuscitated from frozen stocks by first growing overnight on blood agar (Columbia agar plates supplemented with 5% horse blood; Oxoid, Basingstoke, UK) followed by overnight incubation in Brucella broth (Becton, Dickinson and Company, Franklin Lakes, USA). To collect bacteria, cultures were centrifuged at 8000*g* for 5–10 min.

### Cell culture conditions

The HT-29 human colon cancer cell line (ECACC 91072201) was maintained in RPMI 1640 media (Gibco by life technologies, Carlsbad, USA) supplemented with 2 mM glutamin (Swedish National Veterinary Institute, Uppsala, Sweden), 10% fetal bovine serum (FBS, Gibco by life technologies, Carlsbad, USA), 100 U/ml penicillin and 100 µg/ml streptomycin (Swedish National Veterinary Institute, Uppsala, Sweden).

### In vitro cell adhesion assay and IL-8 ELISA

Adhesion to HT-29 cells and IL-8 levels in the media were measured as previously described [30] after inoculation of the cells with bacteria at an MOI of 100. Results are presented as mean and standard deviation of at least two independent experiments.

### RNA preparation from bacteria and cDNA synthesis

Bacterial RNA was extracted from overnight cultures using the ISOLATE II RNA Mini Kit (Bioline Reagents Ltd, London, UK) according to the manufacturer’s protocol. DNase I treatment (Ambion by life technologies, Carlsbad, USA) was performed both on-column and on the final eluted RNA. The concentration of the RNA was determined using Nanodrop and the integrity of the RNA was verified on a 1% agarose gel. One μg of RNA was reverse transcribed using Maxima First Strand cDNA Synthesis Kit for qPCR (Thermo Fisher Scientific, Waltham, USA) according to the manufacturer’s protocol. A minus-RT control reaction was set up for each sample to rule out residual DNA and at least two independent RNA preparations were used. For real-time qPCR, 1/2000 of the cDNA synthesis reaction was used.

### Quantitative PCR

Real-time qPCR was performed in the BioRad CFX96 Touch cycler using the DyNAmo HS SYBR Green qPCR kit (Thermo Fisher Scientific, Waltham, USA). Primer sequences for *C. coli* 16S rRNA and virulence genes are available upon request. For virulence gene expression analyses, expression levels of each gene were first normalized to that of 16S rRNA for each sample and run and thereafter expressed as fold over that of the LMG 6440 reference strain. At least two independent qPCRs were run on each sample.

### Whole genome sequencing

Sequencing of the stool isolates F3, F4 and F8 [30] and the clinical clade 3 isolate 76339 [32] has been described previously. For the blood isolates B5, B49 and B63, DNA was extracted from overnight bacterial cultures using the MagNa Pure Compact Nucleic Acid isolation kit I (Roche, Penzberg, Germany) according to the manufacturer’s protocol version 12. Whole genome sequencing (WGS) was performed at the National Veterinary Institute of Sweden. The libraries for WGS were prepared with a Nextera XT sample preparation kit (Illumina, San Diego, USA). An Illumina MiSeq platform with a 2 x 300 paired end run was used for whole genome sequencing. The single reads were assembled to contigs with Velvet version 7.0.4 [35] running as plugin in Geneious version 8.1.9 [36].

### Genomic analyses

Whole genome sequences of selected *C. coli* reference strains (n = 2), blood (n = 34), stool (n = 38) and animal (n = 54) isolates (Additional file 1: Table S2) were retrieved from GenBank, National Center for Biotechnology Information (http://www.ncbi.nlm.nih.gov/GenBank/index.html; last accessed Feb 1st, 2019). Accession numbers are shown in Additional file 1: Table S2. All included isolates belonged to the ST-828 clonal complex, which was confirmed from the PubMLST database (https://pubmlst.org/campylobacter/). The whole genome phylogenetic tree was constructed in Geneious, using average nucleotide identity (ANI) to closely related taxa calculated using the Gegenees software version 2.2.1 with a threshold of 20% [37]. Sequence alignments and ORF translations of the virulence genes were done in CLC Main Workbench (Qiagen, Hilden, Germany) using standard settings. Calculations of the prevalence of nucleotide mutations were done manually.

### Statistical analyses

To identify statistically significant differences between blood and stool isolates, the Student’s *t* test (unpaired, two-tailed) was used. Significant differences are indicated with asterisks (*p < 0.05, ***p < 0.001).

## Additional files

**Additional file 1.** Additional tables.

**Additional file 2: Figure S1.** Phylogenetic analysis of whole genome sequences. ANI-based phylogenetic tree based on whole genome sequences showing clade division of the *C. coli* clade 1 human blood, stool and animal isolates (n = 128). The *C. coli* clade 1 reference strains RM2228 and LMG 6440 as well as selected previously published clade 2 and 3 sequences (12) were included for comparison.

## Data Availability

Data for calculations of the distribution of *Campylobacter* isolates is available in the PubMLST database (https://pubmlst.org/campylobacter/). All whole genome sequences are available in GenBank, National Center for Biotechnology Information (http://www.ncbi.nlm.nih.gov/GenBank/index.html) under the accession numbers shown in Additional file 1: Table S2. Primers are available from the authors upon request. All additional data supporting the conclusions of this article is included within the article (and its additional files).
