# Supplementary material for: Differences in virulence gene expression between human blood and stool Campylobacter coli clade 1 ST828CC isolates
Source: Gut Pathog. 2019 Aug 1;11:42. doi: 10.1186/s13099-019-0322-9 (PMC6669978; doi:10.1186/s13099-019-0322-9)
Supplement: Supplementary file 1 — Additional file 1. Additional tables. [file 13099_2019_322_MOESM1_ESM.docx]

# ADDITIONAL FILE 1

**Table S1.** Numbers and percentages of *C. jejuni* and *C. coli* isolates in the PubMLST database.

| **Source** | ***C. jejuni*** | ***C. coli*** | ***C. coli***  **ST828CC**  **(clade 1)** | ***C. coli***  **ST1150CC**  **(clade 1)** | ***C. coli***  **Unknown CC**  **(clades 1, 2 and 3)** |
| --- | --- | --- | --- | --- | --- |
| All (n=71559) | 59,508  83.2% | 11,800  16.5% | 8,814  74.7% | 452  3.8% | 2,534  21.5% |
| Human (n=38162) | 34,687  90.9% | 3,335  8.7% | 2,891  86.7% | 25  0.7% | 419  12.6% |
| Blood (n=212) | 151  71.2% | 55  25.9% | 40  72.7% | 0  0% | 15  27.3% |
| Stool (n=34765) | 31,544  90.7% | 3,090  8.9% | 2,696  87.2% | 22  0.7% | 372  12.0% |
| Unknown human  source (n=3185) | 2,992  93.9% | 190  6.0% | 155  81.6% | 3  1.6% | 32  16.8% |

**Table S2.** List of *C. coli* clade 1 isolates of ST828CC included in genomic analyses (n=128) with GenBank accession numbers and the status of the *cdtA*, *cdtB* and *cdtC* open reading frames (+, intact; - disrupted by mutation).

| **Source** | **Strain** | **Accession number** | **CdtA** | **CdtB** | **CdtC** |
| --- | --- | --- | --- | --- | --- |
| Reference (pig) | LMG6440 | QZCI01 | **+** | **+** | **-** |
| Reference (chicken) | RM2228 | AAFL01 | **+** | **+** | **+** |
| Human blood | B5 | SWLU00 | **+** | **+** | **+** |
|  | B49 | SWLV00 | **-** | **+** | **+** |
|  | B63 | SWLW00 | **+** | **-** | **+** |
|  | H044660164 | FBKS01 | **+** | **+** | **-** |
|  | H051080182 | FBOQ01 | **+** | **+** | **+** |
|  | H053280346 | FBOY01 | **+** | **+** | **-** |
|  | H054900335 | FBQT01 | **+** | **+** | **-** |
|  | H060260417 | FAXS01 | **-** | **+** | **-** |
|  | H063540531 | FBNJ01 | **+** | **+** | **-** |
|  | H063900401 | FBKM01 | **-** | **-** | **-** |
|  | H070680142 | FBNW01 | **+** | **+** | **-** |
|  | H073180384 | FBJW01 | **-** | **+** | **-** |
|  | H074360315 | FBOX01 | **+** | **+** | **+** |
|  | H084040382a | FBJY01 | **-** | **-** | **+** |
|  | H084040382b | FBPU01 | **-** | **+** | **-** |
|  | H090780151 | FBOK01 | **+** | **+** | **+** |
|  | H092660305 | FBNL01 | **-** | **+** | **-** |
|  | H093960099 | FBPM01 | **+** | **+** | **+** |
|  | H095340114a | FAZE01 | **+** | **+** | **+** |
|  | H095340114b | FBKC01 | **+** | **+** | **+** |
|  | H103600372 | FBNJ01 | **-** | **+** | **-** |
|  | H104860240 | FAXW01 | **+** | **+** | **+** |
|  | H112820480 | FBPF01 | **+** | **+** | **+** |
|  | H114640463a | FBLE01 | **-** | **+** | **-** |
|  | H123080386 | FBKI01 | **+** | **+** | **-** |
|  | H124620276b | FBPL01 | **-** | **+** | **+** |
|  | H125280575 | FBKU01 | **+** | **+** | **+** |
|  | H130500174 | FBPU01 | **+** | **+** | **+** |
|  | H131800148 | FAYF01 | **+** | **+** | **+** |
|  | H133020651a | FAYK01 | **-** | **+** | **-** |
|  | H133020651b | FBLB01 | **+** | **+** | **-** |
|  | H134460277 | FBPG01 | **+** | **+** | **-** |
|  | H140200373 | FBLY01 | **+** | **-** | **+** |
|  | H142080277 | FBPH01 | **+** | **+** | **+** |
| Human stool | 15-537360 | CP006702.1 | **+** | **+** | **-** |
|  | BIGS00019 | ANHD01 | **+** | **-** | **-** |
|  | F3 | QYUT01 | **+** | **+** | **-** |
|  | F4 | QYUS01 | **-** | **+** | **-** |
|  | F8 | QYUU01 | **-** | **-** | **-** |
|  | FB1 | CP011015.1 | **+** | **+** | **+** |
|  | OXC6253 | CUIX01 | **-** | **-** | **-** |
|  | OXC6258 | CUKZ01 | **+** | **-** | **-** |
|  | OXC6263 | CUHU01 | **+** | **+** | **-** |
|  | OXC6267 | CUHY01 | **+** | **-** | **-** |
|  | OXC6276 | CUII01 | **-** | **+** | **-** |
|  | OXC6297 | CUJD01 | **-** | **+** | **-** |
|  | OXC6308 | CUJQ01 | **-** | **+** | **-** |
|  | OXC6309 | CUJR01 | **+** | **+** | **+** |
|  | OXC6337 | CUKW01 | **+** | **+** | **-** |
|  | OXC6338 | CUKX01 | **+** | **+** | **+** |
|  | OXC6343 | CULD01 | **+** | **+** | **+** |
|  | OXC6371 | CULZ01 | **+** | **+** | **+** |
|  | OXC6372 | CULY01 | **+** | **+** | **+** |
|  | OXC6376 | CUMC01 | **+** | **+** | **+** |
|  | OXC6378 | CUMG01 | **+** | **+** | **+** |
|  | OXC6380 | CUMH01 | **-** | **+** | **-** |
|  | OXC6385 | CUMN01 | **+** | **+** | **+** |
|  | OXC6400 | CUNC01 | **+** | **+** | **+** |
|  | OXC6424 | CUOD01 | **-** | **-** | **+** |
|  | OXC6426 | CUOF01 | **-** | **-** | **-** |
|  | OXC6428 | CUOH01 | **+** | **-** | **-** |
|  | OXC6447 | CUOY01 | **-** | **+** | **-** |
|  | OXC6471 | CUPO01 | **-** | **+** | **-** |
|  | OXC6472 | CUPQ01 | **-** | **+** | **-** |
|  | OXC6513 | CURI01 | **+** | **+** | **-** |
|  | OXC6523 | CURT01 | **+** | **+** | **-** |
|  | OXC6537 | CUSJ01 | **-** | **-** | **-** |
|  | OXC6568 | CUTH01 | **+** | **+** | **+** |
|  | OXC6576 | CUTQ01 | **+** | **+** | **-** |
|  | OXC6577 | CUTR01 | **+** | **+** | **+** |
|  | OXC6601 | CUUO01 | **+** | **+** | **-** |
|  | OXC6630 | CUVQ01 | **+** | **+** | **-** |
| Bovine | 1148 | AIMX01 | **-** | **+** | **-** |
| Bovine | 1909 | AINC01 | **+** | **+** | **+** |
| Pig | BIGS0005 | ANGP01 | **+** | **+** | **+** |
| Pig | BIGS0015 | ANGZ01 | **-** | **-** | **-** |
| Chicken | BIGS0017 | ANHB01 | **+** | **+** | **+** |
| Chicken | BIGS0021 | ANHF01 | **+** | **+** | **+** |
| Chicken | BIGS0024 | ANHI01 | **+** | **+** | **+** |
| Dog | BRISLC31-1 | FBGO01 | **-** | **+** | **-** |
| Pig | BRISPIG3 | FBHR01 | **-** | **-** | **-** |
| Pig | NCTC12568 | FBNI01 | **+** | **+** | **+** |
| Chicken | RC018 | CYPZ01 | **+** | **+** | **-** |
| Chicken | RC023 | CYQC01 | **+** | **+** | **+** |
| Chicken | RC037 | CYQD01 | **+** | **+** | **+** |
| Chicken | RC038 | CYQE01 | **+** | **+** | **+** |
| Chicken | RC043 | CYQF01 | **+** | **+** | **+** |
| Chicken | RC096 | CYQI01 | **+** | **+** | **+** |
| Chicken | RC105 | CYQJ01 | **+** | **+** | **+** |
| Chicken | RC106 | CYQK01 | **+** | **+** | **-** |
| Chicken | RC116 | CYQL01 | **+** | **+** | **+** |
| Chicken | RC126 | CYQM01 | **+** | **+** | **+** |
| Chicken | RC127 | CYQN01 | **+** | **+** | **+** |
| Chicken | RC148 | CYQO01 | **+** | **+** | **+** |
| Chicken | RC182 | CYQT01 | **+** | **+** | **+** |
| Chicken | RC269 | CYQX01 | **+** | **+** | **+** |
| Chicken | RC281 | CYQZ01 | **+** | **+** | **+** |
| Chicken | RC282 | CYRA01 | **+** | **+** | **+** |
| Chicken | RC284 | CYRD01 | **+** | **+** | **+** |
| Chicken | RC285 | CYRE01 | **+** | **+** | **+** |
| Chicken | RC289 | CYRG01 | **+** | **+** | **+** |
| Chicken | RC382 | CYRI01 | **+** | **+** | **+** |
| Chicken | RC383 | CYRJ01 | **+** | **+** | **+** |
| Chicken | RC387 | CYRK01 | **+** | **+** | **+** |
| Chicken | RC415 | CYRO01 | **+** | **+** | **+** |
| Chicken | RC428 | CYRP01 | **+** | **+** | **+** |
| Chicken | SS_2289 | FBFR01 | **-** | **-** | **-** |
| Chicken | SS_2295 | FBDS01 | **+** | **+** | **-** |
| Chicken | SS_2296 | FBEQ01 | **+** | **+** | **-** |
| Chicken | SS_2322 | FBFP01 | **+** | **+** | **-** |
| Chicken | SS_2329 | FBFS01 | **+** | **+** | **-** |
| Duck | SWAN195-3 | FBLI01 | **+** | **+** | **+** |
| Duck | SWAN350 | FBLU01 | **+** | **+** | **-** |
| Duck | SWAN392 | FBLQ01 | **-** | **+** | **-** |
| Pig | UNAJC222 | FBHF01 | **-** | **-** | **+** |
| Pig | UNAJL222 | FBIG01 | **+** | **+** | **+** |
| Chicken | UNCIC2 | FBIW01 | **-** | **+** | **-** |
| Chicken | UNES9 | FBHB01 | **-** | **-** | **-** |
| Chicken | UNES15 | FBHQ01 | **-** | **-** | **-** |
| Chicken | UNF383D | FBGP01 | **+** | **+** | **+** |
| Pig | UNLLIII | FBHV01 | **+** | **+** | **+** |
| Chicken | UNOR10622c | FBMD01 | **-** | **+** | **-** |
| Chicken | UNOR13691b | FBMF01 | **-** | **+** | **-** |
| Chicken | UNOR383B | FBJO01 | **+** | **-** | **+** |
| Chicken | UNOR4451c | FBMH01 | **-** | **+** | **-** |
| Chicken | UNOR532A | FBIJ01 | **-** | **+** | **-** |
