# Supplementary figures and images for: Differences in virulence gene expression between human blood and stool Campylobacter coli clade 1 ST828CC isolates
Source: Gut Pathog. 2019 Aug 1;11:42. doi: 10.1186/s13099-019-0322-9 (PMC6669978; doi:10.1186/s13099-019-0322-9)

Figure S1

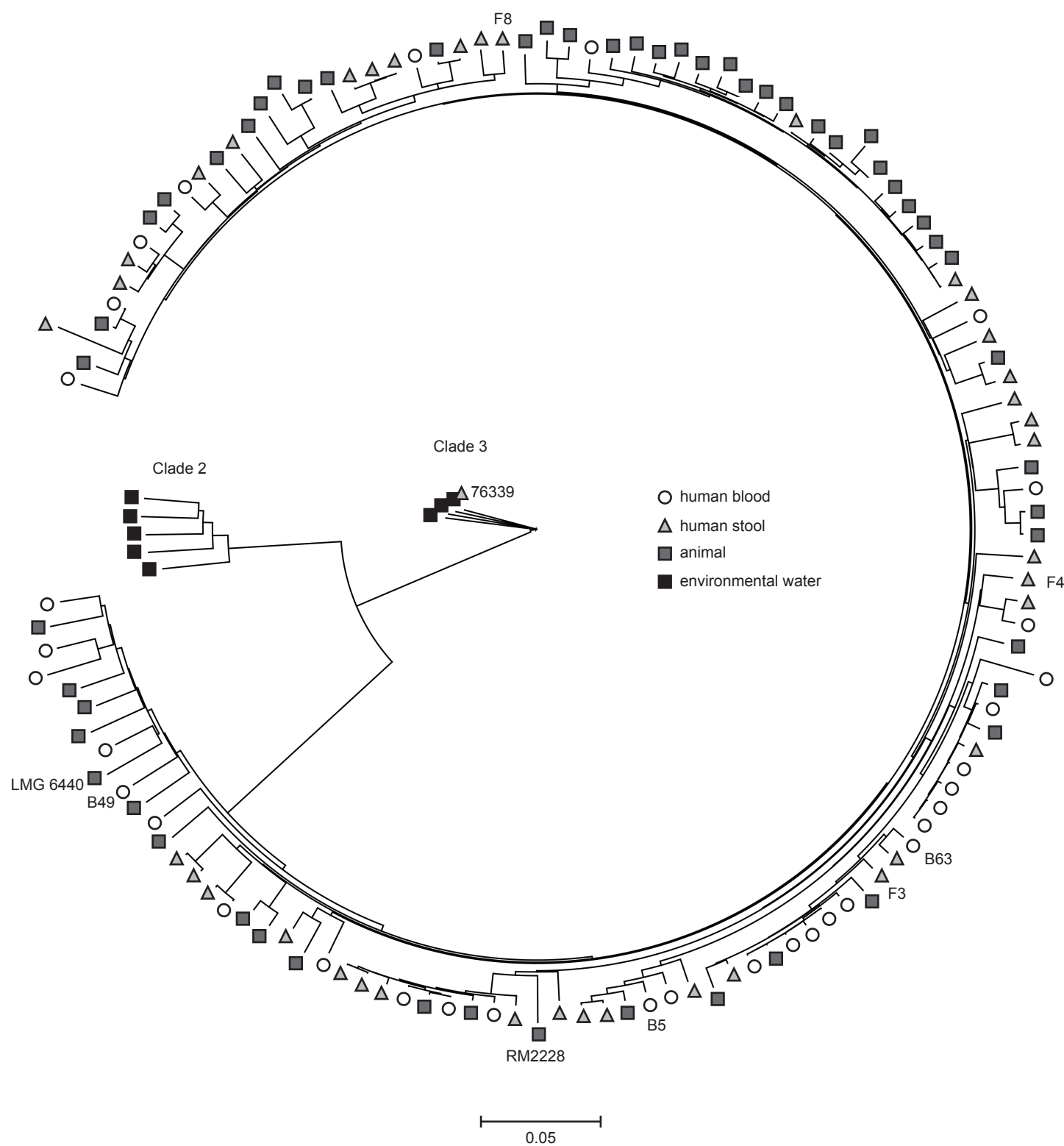

Supplement: Supplementary file 2 — Additional file 2: Figure S1. Phylogenetic analysis of whole genome sequences. ANI-based phylogenetic tree based on whole genome sequences showing clade division of the C. coli clade 1 human blood, stool and animal isolates (n = 128). The C. coli clade 1 reference strains RM2228 and LMG 6440 as well as selected previously published clade 2 and 3 sequences (12) were included for comparison. [file 13099_2019_322_MOESM2_ESM.pdf]
